# Control of Neglected Tropical Diseases in Burundi: Partnerships, Achievements, Challenges, and Lessons Learned after Four Years of Programme Implementation

**DOI:** 10.1371/journal.pntd.0002684

**Published:** 2014-05-01

**Authors:** Onésime Ndayishimiye, Giuseppina Ortu, Ricardo J. Soares Magalhaes, Archie Clements, Johan Willems, Jane Whitton, Warren Lancaster, Adrian Hopkins, Alan Fenwick

**Affiliations:** 1 Programme National Intégré de lutte contre les Maladies Tropicales Négligées et la Cécité (PNIMTNC) - Ministère de la Santé Publique et de la lutte contre le SIDA, Bujumbura, Burundi; 2 Schistosomiasis Control Initiative - Imperial College, Department of Infectious Disease Epidemiology, London, United Kingdom; 3 University of Queensland, Infectious Disease Epidemiology Unit, School of Population Health, Herston, Queensland, Australia; 4 CBM Regional Office for Central Africa, Parklands, Nairobi, Kenya; 5 END Fund/Geneva Global, London, United Kingdom; 6 Mectizan Donation Programme, Decatur, Georgia, United States of America; Centers for Disease Control and Prevention, United States of America

## Introduction

Neglected tropical diseases (NTDs) are a group of 17 conditions that together affect over 1,000,000,000 people in the developing world [1]. Nongovernmental organisations (NGOs) and public-private partnerships have joined efforts to place the NTDs in the spotlight, especially the seven that can be treated with an annual dose of safe and effective drugs: trachoma, three soil-transmitted helminthiases (STHs, ascariasis, hookworm infections, and trichuriasis), lymphatic filariasis (LF), onchocerciasis, and schistosomiasis (SCH) [2]. On the wave of this new interest in NTDs, Burundi was selected in 2007 by the Legatum Foundation for financial support for a national NTD programme.

Before 2007, Burundi hosted two ministerial programmes related to treatment of parasitic diseases: the National Programme for the Control of Transmissible Diseases and Deficiencies (Lutte contre le Maladies Transmissibles et Carentielles [LMTC]), responsible for managing malaria, worm infections, and nutrition, and an onchocerciasis control programme (Programme National de Lutte contre l'Onchocercose [PNLO]). The PNLO, supported by the African Programme for Onchocerciasis Control (APOC) [3] and CBM (formerly known as Christian Blind Mission) [4], involved the annual administration of ivermectin (IVM) in ten endemic districts (namely Cibitoke, Bubanza, Bururi, Rutana, Mpanda, Rumonge, Matana, Gihofi, Mabayi, and the Kayogoro commune in the Makamba district) via the well-established Community Directed Treatment with Ivermectin (CDTI) strategy [5]. For other NTDs, such as trachoma and LF, no specific programmes were in place in Burundi at that time.

Due to limited funding, the LMTC prioritised malaria control activities. Nevertheless, distribution of praziquantel (PZQ) in health centres and mebendazole (MBZ) in schools was already happening between 1973 and 1992 in areas considered highly endemic [6], [7]. Historical epidemiological data on STHs [8] and a survey performed in 2005 [9] suggested that both *Schistosoma mansoni* and STH infections were widespread, with SCH prevalence varying across provinces (range 0–61%, mean 22%) and the overall prevalence of STHs reaching 59.7%.

With the beginning of the civil war in 1993, national prevention and control activities slowed down considerably. Despite the continued political instability, the Ministry of Health (MoH), with UNICEF and the WHO, introduced an annual Mother and Child Health Week (MCHW) in 2003, during which vaccinations, micronutrients, bed nets, and deworming drugs were distributed.

Prior to 2007, trachoma and LF prevalence had never been thoroughly investigated in Burundi. A few cases of trachoma were suspected among refugees, and there was evidence of cases of elephantiasis, one of the clinical manifestations of LF, although they were likely due to podoconiosis [10].

Sparse historical epidemiologic data and the presence of a few early-stage health initiatives (LMTC, PNLO, and MCHW) created an excellent opportunity to roll out an NTD control programme in Burundi. With the specific objectives of defining the population at risk for each infection and elaborating and implementing a drug treatment strategy targeting the population at risk, the final aspiration was to create a solid framework for a sustainable NTD control programme integrated with the health initiatives already in place that would continue after the end of the donor-funded programme.

The present paper provides a critical overview after four years of a programme that started in Burundi in June 2007. Strategies, achievements, challenges, lessons learned, and future steps for the country, especially in relation to schistosomiasis and STHs, are discussed. The onchocerciasis programme coordinated by the national programme is not described in this paper. Details on trachoma that have not been reported already by others [11] are presented as part of the ongoing activities of the NTD programme, as well as the mapping results for LF.

### Assessing the burden of NTDs

NTD mapping was an important step for defining the population at risk of infections and for rolling out drug delivery strategies at the national level.

In 2007, 20 primary schools in the country were randomly selected to assess intestinal schistosomiasis and STHs (using the Kato-Katz method for diagnosis) [12] and urinary schistosomiasis (using the urine filtration method). For LF mapping, because transmission was known to be minimal at altitudes over 1,300 m [12], [13], the NTD control programme mapped LF only in communes situated below 1,500 m. A total of 1,754 adults (aged 15 and above with roughly 100 people per community for a total of 17 communities) were tested via the LF immunocromatographic test (ICT) card [14].

Results of the parasitological surveys confirmed that urinary schistosomiasis and LF were not endemic in the country, whereas intestinal schistosomiasis was highly focalized in areas with close proximity to water bodies. STHs, particularly hookworm, were widely distributed, supporting a mass drug treatment approach targeting nearly the whole population [15]. The enclosed supporting information (Tables S1, S2, S3) outlines the estimated prevalence of worm infections in each commune, with the corresponding risk category as per WHO guidelines.

For trachoma, a rapid assessment was performed [16]–[18], in which an average of 50 adults and 50 children aged 1–9 years per community were examined (n = 2,253 children, n = 1,845 adults). This survey revealed the presence of active trachoma in the country and was followed by a more detailed survey in 2009–2010, in which 20,659 children were examined in 11 districts [11]. Results from this survey demonstrated a prevalence of children with follicular trachoma (TF) above 10% in three districts (Buhiga, Muyinga, and Nyabikere). An extension of this survey to ten other districts also showed TF above 10% in the Rutana district (2013 stakeholders meeting held in Burundi, unreferenced).

**Figure 1:**
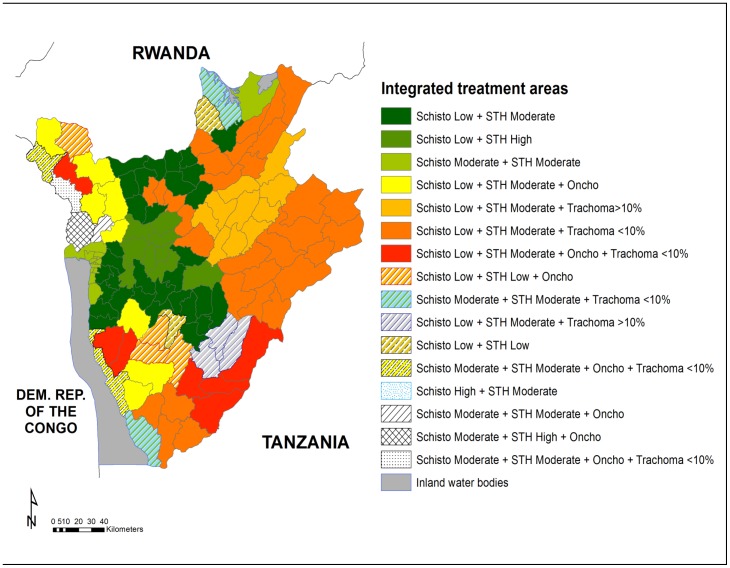
Coendemicity map in Burundi communes in 2010 for trachoma, onchocerciasis, schistosomiasis, and soil-transmitted helminth infections. Coendemicity in Burundi after results obtained from mapping surveys performed in 2007–2010. Colours and patterns indicate presence of disease in cases of onchocerciasis or prevalence of TF below 10% (low) or above ≥10% (high) in cases of trachoma. For schistosomiasis and soil-transmitted helminth infections, colours and patterns indicate disease prevalence, which was estimated via predictive risk maps created with the epidemiological and geospatial integrated approach. In detail, for schistosomiasis, low, moderate, and high indicate prevalence <10%, between 10% and 50%, and above 50%, respectively. For soil-transmitted helminth infections, low, moderate, and high indicate prevalence below 20%, between 20% and 50%, and above 50%, respectively. Abbreviations: Schisto, schistosomiasis; Oncho, onchocerciasis.

All the data obtained were compiled and integrated with environmental and country demographic information to create maps predicting areas at risk of infections (Tables S1, S2, S3) [15] that were then used for defining drug treatment strategies for each mapped disease. Figure 1 shows the coendemicity by commune for trachoma and onchocerciasis in 2010 and the areas estimated at risk of SCH and STHs as per mapping results finalized in 2008. Areas with predicted prevalence estimates ≥10% for SCH, any STH, or TF were considered at risk of infection. Based on these results and on the census performed in 2008 [19], the estimated population at risk was calculated based on an annual growth of 1.03%. Children between 1 and 4 years of age and 5 and 14 years of age and pregnant women between the ages of 15 and 49 represented 14.2%, 26.7%, and 5.0% of the total population, respectively, as estimated by the census. Two thirds of the pregnant women were assumed to be in the 2nd or 3rd trimester of pregnancy.

### Programme implementation

#### Partnerships and leadership

**Figure 2:**
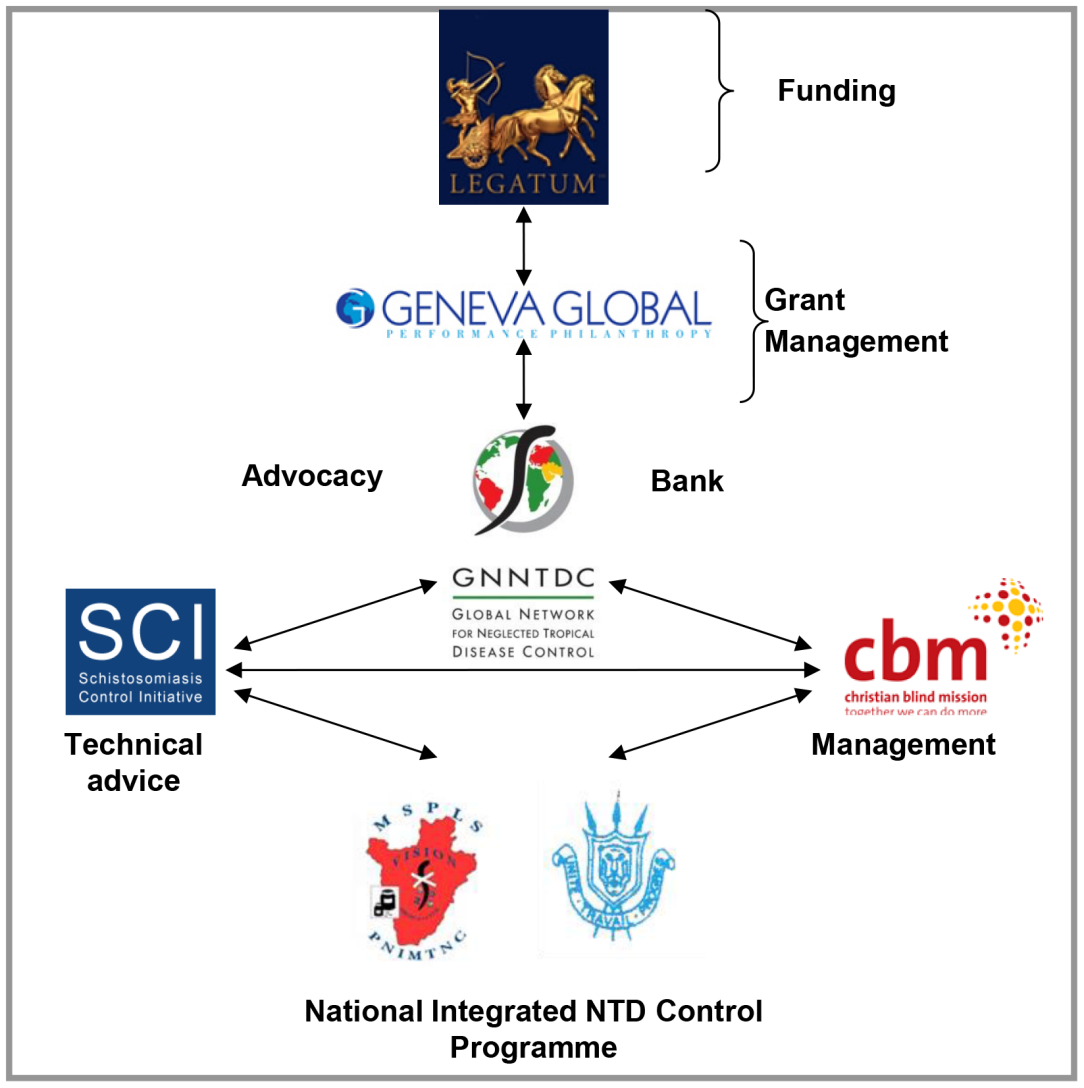
Partners in the NTD control programme in Burundi.

In 2007 the Burundian MoH developed a national NTD working plan and partnered with various organisations for implementation of a four-year programme (2007–2011) (Figure 2). Funding was provided by the investment group Legatum [20] and was channelled through Geneva Global [21], the international philanthropy adviser to the Global Network for NTD Control (GNNTDC) [22], which acted as the primary awarder. The Schistosomiasis Control Initiative (SCI) [23], [24], an NGO which has been running NTD control programmes in Africa since 2003, used its expertise to assist the Burundian MoH NTD control programme in developing action plans. CBM [4], an international NGO that works on preventing and curing disabilities, was subcontracted to assist the NTD control programme by providing management support.

The MoH provided 20 civil servants who constituted the NTD team and office space. The newly formed NTD group represented a focal point within the MoH for consolidating partnerships with other international organizations. These new partnerships facilitated donation of drugs such as MBZ and/or albendazole (ALB) from Feed the Children International, UNICEF, and Food for the Hungry. PZQ was purchased by SCI on behalf of Geneva Global, whereas a partnership with the International Trachoma Initiative led to the donation of azithromycin (AZT).

#### Drug distribution strategies: Options and opportunities

In 2007, the existing CDTI structure had been established in six districts endemic for onchocerciasis, in Rutana, Bururi, and Makamba provinces. In contrast, the MCHW strategy was a national campaign, occurring twice per year, in which vitamin supplementation, vaccinations, and MBZ were distributed to preschool-age children at health posts available throughout the country.

Based on these health structures and the information on NTD coendemicity obtained via the mapping surveys, the MoH piloted an integrated drug distribution in December 2007 in which people in areas endemic for onchocerciasis, SCH, and STHs received IVM, PZQ, and ALB. However, the integrated delivery strategy that used Community Directed Distributors (CDDs) had to be reviewed for the following reasons: (a) distribution of additional drugs was perceived as an increased workload by volunteer CDDs; hence, there was a risk that extra incentives would be requested for delivering additional drugs, and (b) the CDTI network only covered 6 districts at that time, whereas some drugs, such as ALB, had to be distributed at the national level. The creation of a CDTI network for the entire country was not an option as it would have taken many years and the population needed to be treated as soon as possible.

The alternate system that was used in the country, the MCHW, performed better in terms of geographic coverage. However, both drug delivery systems, CDTI and MCHW, did not have school-age children (SAC) as the primary target population in the treatment campaigns. Children between 5 and 14 years of age are the most affected by schistosomiasis and to some extent by STHs, and alternative strategies had to be designed to specifically target this population. One option was to use primary schools as drug distribution points to ensure that SAC were treated in schools during treatment campaigns. For SAC not enrolled in primary schools, possible options were to make PZQ and ALB available in health posts where treatments for children (aged under 5) were already distributed and to create extra distribution points in areas in which health posts were far from villages. To accomplish these treatment goals, the Ministry of Health and the Ministry of Education agreed to endorse PZQ and ALB administration to children in primary schools during the MCHW. During the national campaigns, mobile clinics (“sites avancés”) were also set up to specifically target SAC that were not enrolled in schools and for villages located more than 5 km from health posts.

**Figure 3:**
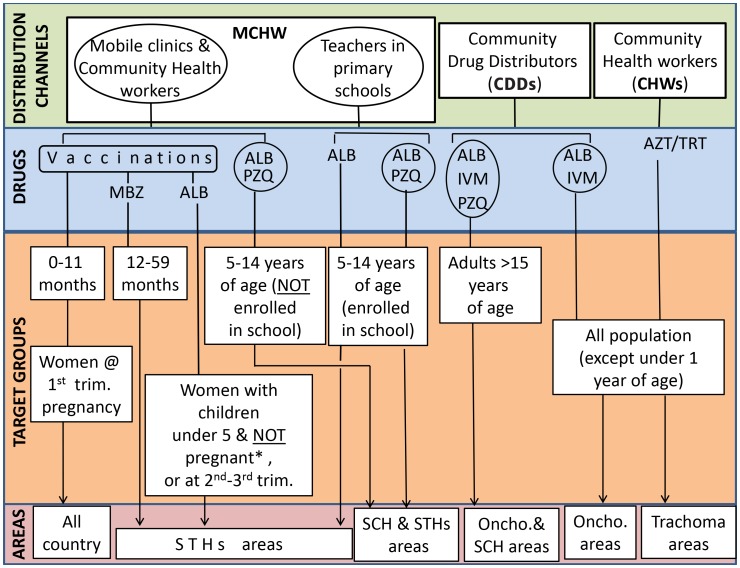
Drug distribution strategies adopted during the NTD programme for trachoma, onchocerciasis, schistosomiasis, and soil-transmitted helminth infections. Distribution channels, drugs (or drug packages), target groups, and endemic areas where drugs were delivered are shown in this figure. Children below 1 year of age and women at the first trimester of pregnancy received vaccinations during the MCHW but no preventive chemotherapy. Adults above 15 years of age in areas at risk of schistosomiasis but not endemic for onchocerciasis (or trachoma) were not targeted by any of the health campaigns and did not receive PZQ. * Considering that no test was available to confirm pregnancy in the first 3 months, women often could not receive ALB if uncertain about their status.

In 2008, the combination of MCHW, primary school treatments, and mobile clinics was embraced as the best-performing strategy for delivering nationwide antihelmintic treatments to children 1- to 4-years-old (MBZ), SAC (PZQ and ALB), and mothers (ALB). In areas endemic for trachoma, azithromycin and tetracycline distribution was done through health workers in villages and in health centres. Distribution of these drugs was not integrated with other NTD treatment campaigns as areas at risk of trachoma were limited; based on the available epidemiological data, it seemed that only one round of treatment was required. Figure 3 summarizes the delivery strategies adopted in Burundi during the NTD programme and their target populations.

#### Rolling out MCHW, school-based, and mobile clinic treatment

Implementation of this innovative strategy involved several steps: (a) setting up a committee to engage partners in financing several activities related to the national mass drug administration campaign and to identify potential financial gaps; (b) training on drug administration procedures; (c) mobilizing the target population with national media, local leaders (e.g., priests and imams), and village chiefs; (d) dispatching drugs to the district and commune levels as well as to schools; (e) launching the mass drug administration (MDA); (f) supervising drug distribution activities to monitor shortages and verify completion of WHO template treatment forms [25]; and (f) evaluating the national programme and geographical drug coverage as per WHO guidelines [26].

On a daily basis in each district, the number of people whose treatment was observed was entered in a database that was sent to a provincial office and finally reported to a central office in charge of combining data from all 45 districts. Programme managers set an expectation of reaching 25% coverage on the first day of the campaign. If coverage was below this expectation, village chiefs were alerted and solicited to sensitize the population.

#### Trachoma treatment and PZQ, IVM, and ALB administration in adults

Treatment for trachoma was limited to those cases that were found positive after the rapid assessment performed in year 2 and the survey performed in year 3. In year 4, the programme distributed azithromycin and tetracycline (TRT) in three of the four endemic districts (Buhiga, Muyinga, and Nyabikere) where TF was found above 10%. A total of 668,719 people were treated, reaching a programme coverage of 91% (calculated with the whole population as target) and a geographic coverage of 75%. For Rutana district, treatment was provided at a later stage (September 2012).

During the NTD programme, CDDs continued IVM treatment in areas with onchocerciasis. In 2007, during the attempt to integrate preventive chemotherapy treatment for onchocerciasis, schistosomiasis, and STHs in adults, a programme coverage of 52% for PZQ (calculated with the whole population as target) and 77% for ALB was achieved in three of the four provinces (Bururi, Cibitoke, and Bubanza) where these diseases were coendemic. The supplementary data files (Tables S1, S2, S3) provide details of the communes where adults were treated with PZQ during this campaign. Fear of secondary effects in the population naïve to PZQ was possibly the reason for the low PZQ coverage in this first integrated administration attempt and for abandoning this strategy in 2008 and 2009.

In 2010, the MoH decided to reorganize a combined ALB+PZQ+IVM treatment campaign in the same provinces plus Rutana. Along with IVM distribution, a total of 621,268 adults and 3,437,164 people were treated with PZQ and ALB, respectively, reaching programme coverage of 114.6% for PZQ and 96% for ALB. Although PZQ and ALB coverage improved in 2010, the combined ALB+PZQ+IVM treatment approach was used only for the adult population in the following years. Treatment of other groups continued via the MCHW+schools+mobile clinics strategy that had been adopted during MDA.

#### ALB and PZQ preventive chemotherapy in mass drug treatments

PZQ administration targeting children between 5 and 14 years of age was done once a year in 29 out of 129 communes, whereas ALB (or MBZ) was administered twice per year in the whole country (45 districts), targeting 1- to 14-year-old children and their mothers (Figure 3).

Following this strategy, Burundi performed one MDA in June 2007 and then one in June and one in December of every year until May 2011 (Table 1). A total of 31,664,642 million treatments were delivered in Burundi during MDAs, of which 2,287,704 were SAC treated for schistosomiasis and 28,700,232 pre-SAC and SAC treated for STHs.

**Table 1:** Individuals treated for schistosomiasis and STHs in each MDA in Burundi.

Target group	2007	2008*		2009*		2010		2011	Total individuals treated in all MDAs
	June	June	Dec	June	Dec	June	Dec	June	
**Schistosomiasis (age group 5–14 y/o)**	N/A	588,214	N/A	545,950	N/A	551,757	N/A	601,783	2,287,704
**STHs (age group 1–4 y/o)**	1,037,169	992,981	1,058,034	3,458,018	1,058,034	1,005,997	3,812,958	1,169,669	28,700,232
**STHs (age group 5–14 y/o**	2,074,183	3,183,872	2,393,429		2,393,429	2,517,275		2,545,184	
**STHs (pregnant women—2nd/3rd trimester)**	98,169	107,940	—	—	122,124	104,466	123,362	120,645	676,706
**Total number of individuals treated each year**	3,209,521	4,873,007	3,559,403	4,003,968	3,573,587	4,179,495	3,936,320	4,437,281	**31,664,642**

* In December 2008 and June 2009, women did not receive treatment.

Table 2 shows the ALB programme coverage at the district level in each target group. Throughout the whole programme, reported coverage in children was above 95% in the majority of the districts; however, some of them reported converges greater than 100%. High coverage reported by the MoH in some districts may be related to children receiving drugs at schools as well as health centres during the same MDA, resulting in double recording of treatment. Furthermore, the wave of unregistered refugees from bordering countries in 2008 increased the total number of people who were treated, inflating the coverage as they were unlikely to have been captured in the estimate of total number at risk.

**Table 2:** Programme coverage for STHs.

	2007^a^		2008^b^				2009^b^				2010^c^				2011^d^	
Target group	June		June		Dec		June		Dec		June		Dec		June	
	Mean	SD	Mean	SD	Mean	SD	Mean	SD	Mean	SD	Mean	SD	Mean	SD	Mean	SD
**STHs (1–4 y/o)**	95.1	11.4	92.8	30.0	98.5	26.6	108.0	35.7	95.6	25.8	81.8	14.6	108.3	16.8	98.6	41.8
**STHs (5–14 y/o)**	98.9	19.5	160.0	44.2	118.5	30.5			115.1	29.6	109.1	12.9			114.5	51.1
**STHs (pregnant women—2nd/3rd trimester)**	38.0	13.3	43.1	18.8	-	-	-	-	47.5	22.3	36.1	12.5	42.9	9.2	42.0	18.1

Means and standard deviations (SD) are calculated based on district programme coverage reported by the Ministry of Health for each district after every MDA. Programme coverage was calculated as the proportion of a specific group of individuals that received ALB over the total number of individuals within that group in the implementing unit deemed at risk of STH infection [26].

In December 2008 and June 2009, women were not treated. Geographic coverage for ALB was always 100%.

a In 2007 coverage was reported by province (n = 17).

b Districts Nyabikere, Gahombo, Busini, and Vumbi did not exist as administrative units in 2008–2009 (n = 41).

c New administrative system included 45 districts (n = 45).

d Women's coverage was not reported in June 2011 for Kayanza district.

A total of 676,706 women in the 2nd and 3rd trimester of pregnancy were given ALB during the MCHW, reaching programme coverage between 35 and 47% during the campaigns (Tables 1–2). This low coverage could possibly be explained by the fact that during prenatal consultations, women diagnosed with worm infections were often advised not to take any drug during pregnancy. International guidelines supporting helminth treatment in pregnant women [27], [28] encouraged the government to offer ALB as a blanket treatment to pregnant women for free during the MCHW campaign. However, during this campaign, women were free to make the final decision on either taking ALB free of charge (irrespective of their infection status) or paying a fee for treatment at the health centre if infection was found after the pregnancy was over. Currently the MoH is looking into new strategies to sensitise pregnant women to deworming activities.

For PZQ, treatment was limited to 29 communes at risk of infection (Tables S1, S2, S3). Because treatment decisions were made at the commune level and the number of people treated was reported by district, it was not possible to calculate the geographic coverage or the programme coverage for each commune every year. From personal communications, reported coverage at the national level for PZQ administration in school-age children was 87.9%, 92.2%, 114.6%, and 95.9% in MDAs performed in the years 2008–2011, respectively; however, it was not clear whether this coverage was calculated based on the targeted population at the commune level or at the district level.

## Discussion

### Opportunities, challenges, and lessons learned

**Figure 4:**
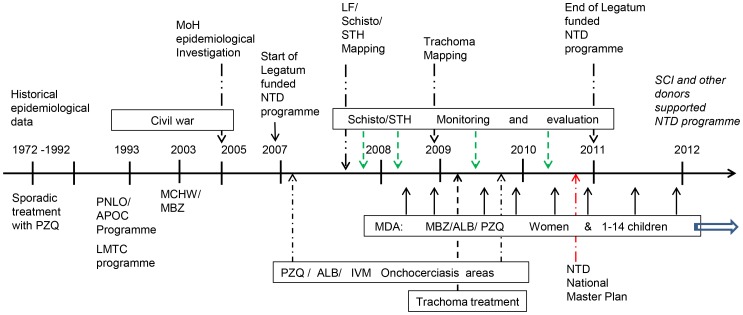
Evolution of the NTD prevention and control programme in Burundi.

Despite the sociopolitical instability, Burundi has gradually developed a structured NTD control programme. Figure 4 summarizes the various stages of this programme. From two vertical programmes focusing on onchocerciasis and malaria, Burundi recognized the importance of addressing other NTDs affecting the population and the need to integrate their prevention and control with health care initiatives already in place in the country, an approach that has already been proven by others to be more sustainable in the long run [29]–[31]. The combination of the national Expanded Programme on Immunization via MCHW with deworming drug delivery, the intersectoral collaboration with primary schools, and the use of mobile clinics during the MCHW appeared to be a very successful strategy that allowed all target groups to be reached and confirmed the feasibility of integrating specific health programmes (such as the NTD control) with primary health care initiatives. To the best of our knowledge, only Rwanda applied a similar drug administration strategy in those years, when the MoH, in collaboration with the Access Project [32] and supported by the same donors [33], started its NTD prevention and control programme.

It should be noted that treatment frequency and areas targeted by the distribution did not always follow the standardized international treatment guidelines [27] or the treatment strategies suggested by the mapping results. For instance, for schistosomiasis treatment, according to the mapping in 2008, 24 out of 129 communes had a predicted prevalence of schistosomiasis between 10 and 50%. This suggested that in those communes SAC and at-risk adults should have received PZQ every two years. However, the government opted for a more intense approach with a higher frequency of treatment, which seemed to be more suitable in the national context considering that (a) MCHW were performed twice a year so the addition of one or more drugs did not substantially increase the cost of this campaign, and (b) poor access to clean water and sanitation were and still are major factors contributing to reinfection.

Another example is treatment of adults in onchocerciasis and schistosomiasis coendemic areas. This group was treated with PZQ in coendemic areas in five communes where the schistosomiasis estimated predicted mean prevalence was above 10%, as well as in eight of those in which this prevalence was below 10%. PZQ administration in these eight communes was not a decision driven by the schistosomiasis disease burden but by the CDTI structure already in place, and it was based more on logistic considerations than epidemiological reasoning. The risk of using logistic considerations for drug distribution may lead, as in this case, to missing PZQ administration in areas of the country where only schistosomiasis is endemic or delivering unneeded PZQ in areas at low risk of schistosomiasis because a distribution structure is already in place. Considering that the global PZQ production is based on the number of people in high-risk areas in need of treatment, drug delivery in areas at low risk of infection might adversely affect PZQ availability for priority groups and people at high risk of schistosomiasis.

Finally, areas identified for PZQ distribution were also adjusted according to the current changing context. Fifteen communes with a predicted mean prevalence >10% and 14 with prevalence of <10% (Tables S1, S2, S3) were also treated with PZQ, leaving nine communes that were at medium predicted risk with no treatment based on the improved urban setting of these communes (e.g., the relatively good socioeconomic conditions) and environmental factors such as the absence of the snail host and stagnant water.

For STHs, the MoH decided again to administer more ALB than international guidelines suggest: seven, ten, and 28 districts out of 45 had a predicted prevalence above 50%, between 20 and 50%, and between 10 and 20%, respectively (Tables S1, S2, S3). Based on these estimates and on the WHO guidelines available at that time [27], the country should have delivered ALB in only seven districts twice a year and ten districts once a year, with other districts adopting a case management approach.

The programme presented challenges from which we have learned important lessons. For instance, the programme did not include a thorough supervision at the lowest implementation level: NTD educational material that reached health units did not reach primary schools; likewise, laboratory technicians in decentralized health centers that were trained in the Kato Katz method for detection of worm infections never received the material for running this test. Better supervision at the lowest level of the programme implementation would have ensured that the primary beneficiaries were properly reached and that the main goals of these activities (education on NTDs for SAC in the first case and detection and treatment of infected people in the second example) would have been achieved.

Finally, Burundi identified the commune, and not the district, as the implementing unit for distributing PZQ. Although this strategy appeared to be very appropriate, reported coverage calculated at the district level did not allow a rapid detection of communes that could have been missed by the MDA or perhaps did not require MDA. It would have been more effective to report programme coverage according to the actual implementing unit (commune) to verify in real time possible mistakes in drug distribution and implement the correct measures.

### Looking ahead

Burundi benefitted greatly from this NTD programme, which has resulted in the mapping of the four major NTDs (schistosomiasis, STHs, LF, and partially trachoma), delivery of treatments reaching the population at risk of infections with good coverage, and a five-year national plan for NTD prevention and control endorsed by WHO and validated by the MoH. The programme has also demonstrated that the integration of NTD treatments with other health initiatives, such as the Expanded Programme of Immunization and the use of mobile clinics plus primary schools as additional distribution sites, is a powerful strategy for reaching the whole population at risk. With the current drug donation programs [34], [35] and the continuous financial and technical support from international NGOs, especially from UNICEF for the MCHW campaigns, Burundi is in a position to complete the mapping of trachoma in the remaining 24 districts and to continue MDAs for the major NTDs affecting the population.

For the sustainability of this NTD programme and with the final aim of eliminating these diseases, the initiation of a multidisciplinary approach involving other ministries, such as those responsible for water and sanitation, is paramount. All these intersectoral initiatives could be effectively coordinated by the government to ensure their long-term sustainability. Coordination and effective management of intersectoral initiatives could be achieved by establishing technical groups within the MoH that would be responsible for linking specific NTD control needs with other initiatives relevant to this programme (e.g., water and sanitation initiatives). Delegation of these groups for all technical aspects of the NTD management and divulgation of intersectoral work plans and technical material would be the next steps to achieve an effective coordination of multidisciplinary activities. These steps will imply a shift towards complete ownership of the NTD programme by the MoH.

Meanwhile, effort should be put into the development of an efficient decentralized routine surveillance system within the primary health care structure for effective case detection, management, and follow-up care. This step should be taken in Burundi when disease prevalence has been reduced and case management replaces mass drug administration. After initial technical and financial support from international stakeholders for building NTD surveillance capacity, financial commitment of the MoH to maintain this structure in decentralized areas will ultimately be expected for sustainability of the NTD prevention, control, and surveillance program in Burundi.

## Supporting Information

Table S1 **Burundi administrative units in year 2012.** Administrative units in Burundi updated to year 2012 (provinces, districts, and communes).
(XLSX)

Table S2 **Estimated risk prevalence of schistosomiasis in each commune and treatment by commune in 2007–2011.** Epidemiological data obtained in 2007 during the mapping were compiled and integrated with environmental and country demographic information to obtain estimated mean prevalence of schistosomiasis in each commune. Based on WHO guidelines (2006), communes were classified as high, moderate, and low risk of infection. These data were then used to create maps predicting areas at risk of schistosomiasis (see Figure 1). The table also provides information about praziquantel treatment and the population targeted in each commune.
(XLSX)

Table S3 **Estimated risk prevalence of soil-transmitted helminth infections in each district.** Epidemiological data obtained in 2007 during the mapping were compiled and integrated with environmental and country demographic information to obtain estimated mean prevalence of soil-transmitted helminth infections in each district. Based on WHO guidelines (2006), districts were classified as high, moderate, and low risk of infection. These data were then used to create maps predicting areas at risk of soil-transmitted helminth infections (see Figure 1). The table also provides information about suggested drug strategies for each district based on predicted risk and on WHO guidelines available in 2006.
(XLSX)
